# Editorial: Molecular influences in therapies in ovarian cancer

**DOI:** 10.3389/fonc.2022.991769

**Published:** 2022-08-08

**Authors:** Stefano Restaino, Jian-Jun Wei, Giuseppe Vizzielli, Fabio Martinelli

**Affiliations:** ^1^ Department of Maternal and Child Health, Obstetrics and Gynecology Clinic, University-Hospital of Udine, Udine, Italy; ^2^ Department of Pathology, Northwestern University, School of Medicine, Chicago, IL, United States; ^3^ Department of Medicine, Obstetrics and Gynecology Clinic, University of Udine, Udine, Italy; ^4^ Fondazione Istituto Nazionale Tumori Istituto di Ricerca e Cura a Carattere Scientifico (IRCCS), Milan, Italy

**Keywords:** ovarian cancer, therapy, treatment, epithelial mesenchymal transition, metastatic, molecular

Ovarian cancer is one of the most common gynecological malignancies leading to one of the highest causes of cancer-related deaths in women worldwide. Approximately over 250,000 women are diagnosed globally and over 150,000 patients pass due to this disease annually (1).

Surgical treatment is typically performed for early ovarian cancer to remove the tumor and make a definite diagnosis and to classify the stage of the disease (2). Cytoreductive surgery combined with platinum-based chemotherapy is commonly used for advanced ovarian cancer patients and despite the short-time effect, approximately 70% of patients suffer from recurrence after first-line treatment which negatively impacts survival time and prognosis (2, 3). Ovarian cancer requires the identification of potential therapies and treatments for ovarian cancer to improve the survival rate and prognosis of the disease. However, studies have demonstrated that therapies and treatments can be influenced by molecular mechanisms that need to be further studied. For example, several studies have found that an active therapeutic target for combination treatment was found to be the DNA damage response pathway, such as Poly (ADP-ribose) polymerase (PARP) (4).

It is evident how the role of molecular biology is crucial to define a individualized treatment in patient affected by ovarian cancer.

It was an honor and a pleasure for us to serve as Guest Editors of the Research Topic of Frontiers entitled “*Molecular Influences in Therapies in Ovarian Cancer*”. We are pleased to present a series of articles produced by proven experts in the field of gynecology and oncology. All authors that contributed to the Research Topic are authors contributing to important advancements in clinical and basic research. This Research Topic provides an overview about molecular influences in ovarian cancer.

We are of the opinion that the topics discussed in this Research Topic will be of relevance to a wide audience, from basic academic researchers to clinicians in gynaecology, oncologists, geneticists and surgeons.

The Research Topic opens with an original artcile by Yan et al., entitled “*The Overexpression of Acyl-CoA Medium-Chain Synthetase-3 (ACSM3) Suppresses the Ovarian Cancer Progression via the Inhibition of Integrin β1/AKT Signaling Pathway*”. The authors observe that Acyl-CoA medium-chain synthetase-3 performs as a tumor suppressor gene and may be a potential therapeutic target of ovarian cancer. Shen et al. demonstrate the importance of Intraoperative Frozen Sections and the accuracy in the diagnosis of ovarian tumors. An interesting review and meta-analysis by Han et al. with the aim to evaluate the value of serum Human epididymis protein 4 (HE4) for predicting the resistance of ovarian cancer (OS) to platinum chemotherapy. Ren at al. produced a systematic review and meta-analysis about the role of PARP in combination with other drugs. The authors observe an increase of PFS in patients with recurrence ovarian cancer. No doubt the topic regarding the use of PARP inhibitors played a predominant role. Qian et al. have investigated the role of PARP inhibitor in patients with Pathogenic Germline FANCA-Mutated Relapsed Epithelial Ovarian Cancer. Several studies have focused on the role of different biomarkers in ovarian tumours (Qian et al., Yang et al., Li et al., Xiong et al., Yang et al., Wang et al., Wang et al., Zhang et al., Li et al., Guo et al.). Chen et al. have highlighted and emphasized how the role systematic lymph node dissection may be reviewed in patients with apparent early-stage low-grade mucinous and endometrioid epithelial ovarian cancer but may be considered for apparent early-stage low-grade serous patients. Hou et al. try to determine the risk and prognostic factors of ovarian cancer in women submitted to fertility-sparing surgery (Huo et al.). The authors conclusions are: “the constructed nomograms exhibited superior prognostic discrimination and survival prediction for patients with stage I epithelial ovarian cancer”. The last article by Huo et al. entitled *“FAK PROTAC Inhibits Ovarian Tumor Growth and Metastasis by Disrupting Kinase Dependent and Independent Pathways*” evaluating the role of FAK PROTAC inhibits both FAK kinase activity and its scaffold protein activity by disrupting the interaction between FAK and ASAP1 and is highly effective in inhibiting ovarian tumor growth and metastasis.

We would like to sincerely thank all the authors for these high-quality and valuable articles that contribute considerably to the scientific panorama.

## Author contributions

SR, J-JW, GV, FM: writing, literature search. All authors: reviewing of the final manuscript. All authors contributed to the article and approved the submitted version.

## Conflict of interest

The authors declare that the research was conducted in the absence of any commercial or financial relationships that could be construed as a potential conflict of interest.

## Publisher’s note

All claims expressed in this article are solely those of the authors and do not necessarily represent those of their affiliated organizations, or those of the publisher, the editors and the reviewers. Any product that may be evaluated in this article, or claim that may be made by its manufacturer, is not guaranteed or endorsed by the publisher.
